# Global functional diversity of freshwater fish is concentrated in the Neotropics while functional vulnerability is widespread

**DOI:** 10.1038/srep22125

**Published:** 2016-03-16

**Authors:** A. Toussaint, N. Charpin, S. Brosse, S. Villéger

**Affiliations:** 1 CNRS, UPS, ENFA, UMR 5174 EDB (Laboratoire Évolution et Diversité Biologique), Université Paul Sabatier, 118 route de Narbonne, F-31062 Toulouse, France; 2 Laboratoire Biodiversité Marine et ses Usages (MARBEC), UMR 9190 CNRS-UM-IFREMER-IRD, Université de Montpellier, CC 093, F-34095 Montpellier Cedex 5, France

## Abstract

Worldwide biodiversity assessments have mainly focused on species richness but little is known about the diversity of species roles, i.e. functional diversity, while this is a key facet to understanding the consequences of global changes on the ecosystem services to human societies. Here, we report the world pattern of functional diversity of freshwater fish using a database encompassing morphological characteristics of more than 9,000 species. The Neotropical realm hosts more than 75% of global functional diversity while other realms each host less than 25%. This discrepancy is mediated by high functional uniqueness in some diversified Neotropical fish orders. Surprisingly, functional diversity patterns were weakly related to functional vulnerability. In the Neotropics the loss of threatened species will cause a limited loss of functional diversity (<10%) whereas in the Nearctic and Palearctic realms, decline of the functional diversity will reach 43% and 33%, respectively, conferring a high functional vulnerability to these realms. Conservation of the Neotropical fish diversity is a key target to maintain world fish functional diversity, but this should not hide the pressing need to conserve the vulnerable fish faunas of the rest of the world, in which functional diversity is to a large extent supported by threatened species.

Owing to the sixth mass extinction crisis^1^, colossal efforts have been devoted to assessing biodiversity, but to date, most worldwide biodiversity assessments have focused on the taxonomic component of biodiversity^2^,^3^,^4^,^5^. These worldwide assessments on taxonomic diversity have revealed that species richness varies strongly across the world for most clades, e.g. birds^5^, plants^6^ or fishes^7^, with a decreasing richness gradient from the equator to the poles^2^. For the freshwater fish fauna, the Neotropical realm (South and Central Americas) includes more than 4,000 fish species, while the Palearctic realm (Eurasia, Middle-East and North-Africa) contains fewer than 600^8^. Besides this taxonomic facet, functional diversity (FD, i.e. the range of biological traits^9^) is a measure of the range of functions performed by organisms, and therefore a good way to approach the role of biodiversity in sustaining ecosystem services^9^,^10^,^11^,^12^,^13^,^14^,^15^, as well as the detrimental effects of human disturbances on the erosion of biodiversity^12^. FD is expected to increase with taxonomic diversity (TD) because of the higher probability of including a larger range of biological traits when more species are present^16^,^17^, but the influence of TD on FD is still unclear, as the lack of large-scale assessments of functional traits on a large range of species limits our capacity to run global scale studies on FD^18^,^19^.

Here, we assess for the first time the FD of the native freshwater fish faunas over the 6 terrestrial biogeographic realms of the world. Functional diversity was measured as morphological diversity^20^,^21^. Although these traits are not all directly linked to the actual roles played by fish in ecosystems (e.g. nutrient recycling^22^, trophic control of other taxa^23^), they remain informative to describe features related to food acquisition and locomotion^24^,^25^,^26^, and remain the only traits measurable for almost all fish species with a reasonable effort. We here measured 10 morphological traits on 9,170 fish species (see Supplementary Fig. S1 for details).

We quantified the extent to which FD, calculated as the volume filled by the species in the multidimensional space defined by morphological traits, differs between the biogeographic realms. Then, we tested the contribution of each fish order to each realm’s functional diversity by measuring the functional uniqueness of each order within the realm (F_uniq_, i.e. proportion of the functional space of the realm filled only by this order). Finally we assessed functional vulnerability to species loss as the proportion of FD supported by species listed as ‘threatened’ by the IUCN^27^ (i.e. status is “critically endangered”, “endangered” or “vulnerable”), and by species endemic to a single river drainage basin and not listed as “least concern” or “near threatened” by the IUCN^27^,^28^.

## Results and Discussion

### Neotropics host more than three-quarters of the world fish functional diversity

**Figure 1:**
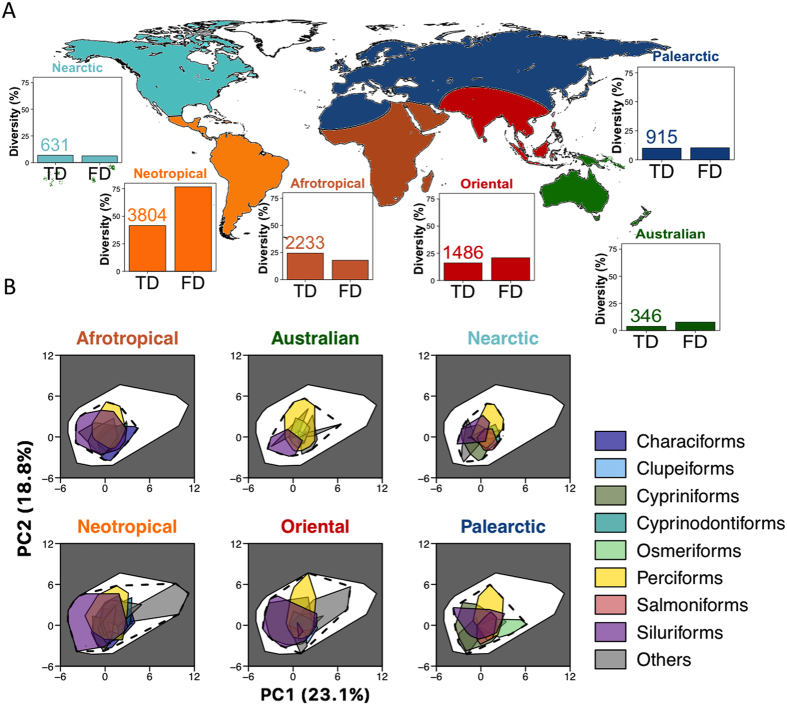
Fish taxonomic and functional diversity in the 6 biogeographic realms. (**A**) The six realms are displayed on the map. For each realm, the taxonomic (TD) and the functional diversity (FD) are represented as a percentage of the world taxonomic or functional diversity. The number of species in each realm is given above the TD bar. (**B**) Functional space of the world freshwater fishes is the white area in the 2-dimensions space made by first and second principal component (PC) axes summarizing the 5-dimensions functional space built based on 10 morphological traits (see other projections in Supplementary Fig. S2). The percentage of variance explained by each PC axis is given in brackets (see also Supplementary Figs S2 and S3). Fish functional diversity in each realm is illustrated by the polygon delimited by the black dashed line. The coloured polygons within it show the functional diversity of the most speciose fish orders. The map was generated using ‘maps’ and ‘mapproj’ libraries, available in R 3.0 (http://www.r-project.org).

We report a threefold lower functional turnover (mean ± SD: 0.324 ± 0.206) than taxonomic turnover between realms (0.982 ± 0.022). Thus, despite strong species turnover between realms, the same core of biological attributes is shared between realms (Fig. 1B, Supplementary Fig. S2). Such a pattern of functional nestedness between realms hosting different suites of species has been reported for two other vertebrate taxa, passerine birds^21^ and coral reef fish^19^.

**Figure 2:**
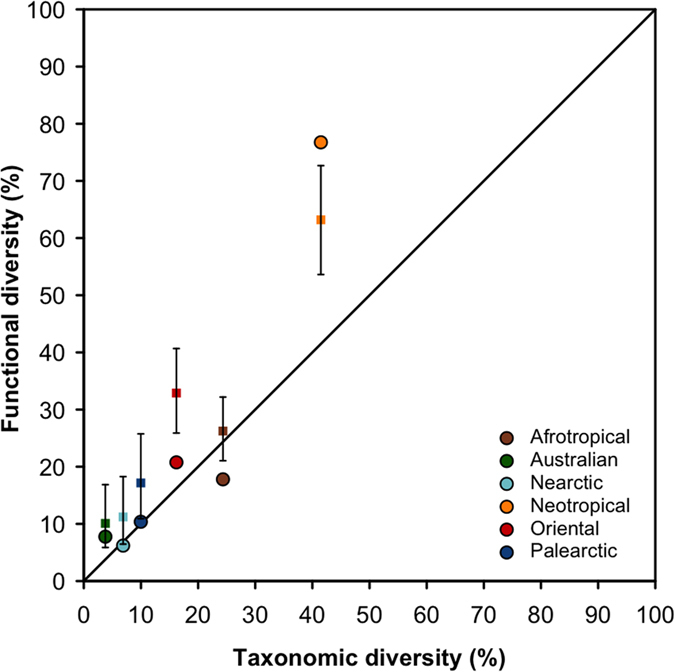
Taxonomic and functional facets of fish biodiversity in the 6 biogeographic realms. Functional diversity (FD) and taxonomic diversity (TD) are expressed as a percentage of the world functional and taxonomic diversity of freshwater fish. The effect of species identity on functional diversity is represented by the mean (colored squares) and associated 95% confidence interval (black whiskers) computed on 999 values of a null model. This null model simulated realm assemblages using random sampling in the world pool of species, while conserving the taxonomic structure of the realm fauna (i.e. number of species per order). Observed values of FD above the whiskers indicate a significant functional overdispersion, whereas values below the whiskers indicate significant functional clustering. The solid line represents the identity line FD = TD. Each color refers to one realm.

Besides this shared functional core, we show here a clear spatial discrepancy of fish FD among realms. The Neotropics host more than three-quarters of the world FD (76.7%) whereas the two other tropical speciose realms, namely the Oriental and the Afrotropical realms, each hosts only one-fifth (17.8% and 20.8%, respectively, Fig. 2). Hence, the twofold higher TD in Neotropics compared to the Afrotropics turns into a fourfold higher FD. A similar trend was observed between Neotropical and Oriental realms, confirming that the Neotropical fauna is functionally hyperdiverse (Fig. 2). Hence, the Neotropics, which represent less than 15% of the world’s continental surface, host more than 75% of the world freshwater fish FD. In contrast, it is surprising to note that the FD of the Afrotropics is close to that of the Palearctic realm, while the latter hosts less than half as many species^8^. Similarly, the Australian realm hosts a FD close to that of the Nearctic although it only hosts half as many species (Figs 1A and 2).

The weak association we report between TD and FD suggests that the diversity of species traits present in a realm is not a random subset of the pool of traits values at the world scale. Indeed, we found that the Neotropical fish fauna has a significantly higher FD than expected given its TD (Standardized Effect Size (SES) = 2.21, *P* > 0.99) whereas the FD of Afrotropical (SES = −2.46, *P* < 0.01) and Oriental realms (SES = −2.50, *P* < 0.01) were significantly lower than expected (Fig. 2). FD did not differ from random expectation for Australian fauna (SES = −0.79, *P* = 0.22) whereas a significantly lower FD was reported for Nearctic (SES = −1.32, *P* < 0.05) and Palearctic realms (SES = −1.54, *P* < 0.05). The functional overdispersion of the Neotropical fish fauna could be due to (*i*) either a few functionally diversified orders that fill most of the functional space (the remaining functionally poor orders being nested in the functionally rich orders), or (*ii*) a high functional dissimilarity between orders, i.e. each order filled a unique part of the realm FD. On the other hand, the functional clustering of the Afrotropical and Oriental realms could be driven by (*i*) a low functional diversity of all orders and/or (*ii*) a high functional similarity (i.e. redundancy) between orders.

### Differences in functional diversity are driven by the functional uniqueness of a few fish taxa

**Figure 3:**
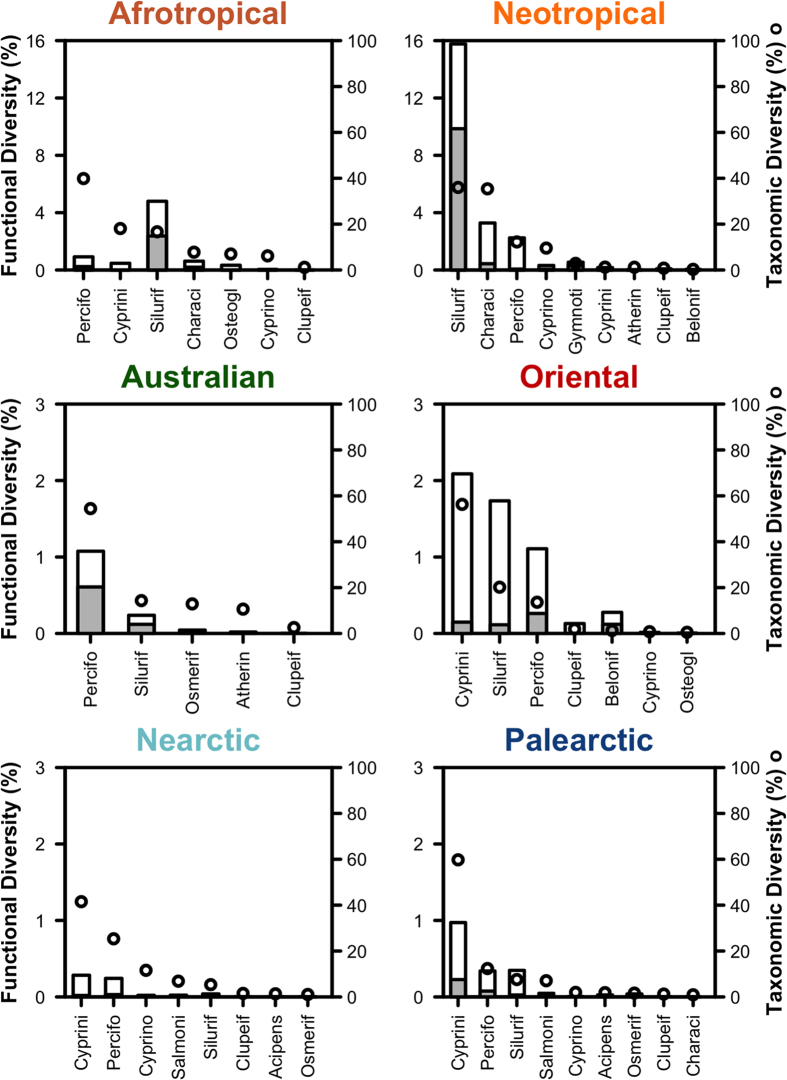
Functional diversity and uniqueness of fish orders within realms. The functional diversity (FD) of each fish order in each realm is given as the percentage of the world FD (bars). The part of FD of each order non-filled by any other order in the realm (Functional uniqueness, F_uniq_) is the ratio between the grey part and the FD of each order. Note that the FD scale differs between realms. The orders are sorted by decreasing TD in each realm and the black dots indicate the percentage of the realm TD represented by each order. Only the 13 most speciose orders in the world are represented (Acipens: Acipenseriforms; Atheri: Atheriforms; Belonif: Beloniforms; Characi: Characiforms; Clupei: Clupeiforms; Cyprini: Cypriniforms; Cyprino: Cyprinodontiforms; Gymnoti: Gymnotiforms; Osteogl: Osteoglossiforms; Osmerif: Osmeriforms; Percifo: Perciforms; Salmoni: Salmoniforms; Silurif: Siluriforms).

Although the FD of orders at the global scale was correlated to their TD (Spearman’s rank correlation rho = 0.31, *n* = 46, *P* < 0.05), the functional uniqueness (F_uniq_) of orders was independent of order TD (Spearman’s rank correlation rho = −0.04, *n* = 46, *P* = 0.76). Indeed, the realm FD results not only from the global FD of each order (Fig. 1), but also from the degree of functional uniqueness between orders (Fig. 3). The functional clustering for all realms except the Neotropics is thus due to the low functional uniqueness of their most speciose orders (Fig. 3). This is particularly striking for the Afrotropics, where the two most speciose orders (Perciforms and Cypriniforms) that together account for more than 60% of the Afrotropical species richness, contribute marginally to the realm FD (5.2 and 2.6%, respectively) because of their low F_uniq_ (26.2 and 0.4%, respectively, Fig. 3). This was verified for the Perciforms, despite the recent and intense radiation of cichlids (the most species rich family of Perciforms in the Afrotropics) promoted by the recent opening of the rift lakes (e.g. Malawi, Tanganyika, Victoria) that produced hundreds of phylogenetically closely related species^29^. Although the great lakes cichlids radiations have generated diverse morphological adaptations^29^, the resulting morphological features did not strongly differ from those already experienced within the African fish fauna, hence explaining the low functional uniqueness of the Afrotropical fish orders.

In contrast, the Neotropical fish fauna hosts species with unique functional attributes such as *i)* extremely elongated fish with a large terminal mouth and a high caudal peduncle throttling, corresponding to mobile surface predators such as some of the Beloniforms, or *ii)* dorso-ventraly flattened fishes, with a ventral mouth located below the head and a small caudal peduncle throttling, mainly corresponding to benthic algae browsers with limited swimming efficiency such as some Loricariid species (see Supplementary Fig. S3). The Loricariids belong to the Siluriforms that is the most speciose order in the Neotropics and they strongly contribute to the high FD of the realm due to their high F_uniq_ (62.5%, Fig. 3). Indeed Neotropical Siluriforms have an impressive body size range (from less than 5 cm to more than 200 cm), a wide span of diet (from algae browsing in the Loricaridae, to ichthyophagy in the Pimelodidae, and even parasitism in the Trichomycteridae, see Supplementary Fig. S3), and of habitat associated with diverse body shapes from flattened to extremely elongated^30^. Such a high FD of Siluriforms is not observed in the other realms, explaining their low functional uniqueness (6.6% in Oriental, 7.4% in Nearctic and 7.1% in Palearctic realms, Fig. 3) despite their contribution to realm TD (e.g. 373 Siluriforms species in the Afrotropical realm).

### Functional vulnerability peaks in the Nearctic and Palearctic realms, despite their low functional diversity

**Figure 4:**
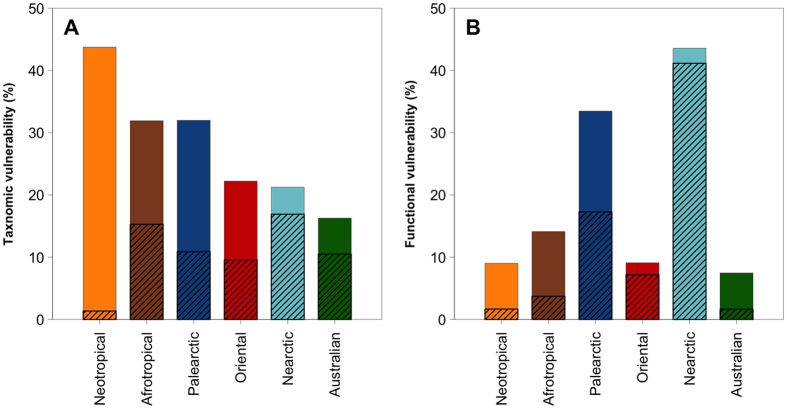
Vulnerabilities of taxonomic and functional diversity to extinction of threatened species. (**A**) Taxonomic vulnerability. Bars represent the proportion of threatened species (i.e. species with an IUCN status “CR”, “EN” or “VU” or species occurring in only one drainage basin across the world which IUCN status was not “Near threatened” nor “Least concern”). The hatched part of each bar represents the taxonomic vulnerability due only to the most threatened species (i.e. the species listed as “CR”, “EN” or “VU” by the IUCN). (**B**) Functional vulnerability. Bars represent the loss of functional diversity (FD) if threatened species become extinct. The hatched part of each bar represents the functional vulnerability due only to the most threatened species (i.e. the species listed as “CR”, “EN” or “VU” by the IUCN).

The loss of FD through species extinction is expected to be marked when threatened species support unique functions, as shown in alpine plants, tropical trees, coral reef fishes and bird regional assemblages^19^,^31^,^32^. For freshwater fish, the vulnerability of FD to the extinction of threatened species differed among realms by a sixfold factor (Fig. 4), and was neither correlated to the realm FD (Spearman’s rank correlation rho = −0.43, *P* = 0.42) nor to the number of threatened species (Spearman’s rank correlation rho = −0.08, *P* = 0.92). The loss of FD due to the simulated extinction of threatened species was marked in the Nearctic and Palearctic realms (43.6 and 33.5%, respectively, Fig. 4), while it was low (from 7.47 to 14.1%) in the Afrotropical, Australian, Neotropical and Oriental realms. Within this last group, the Neotropics are characterized by a vulnerability of FD to species loss lower than expected given the number of threatened species (SES = −2.32*, P* < 0.001, Table 1). In contrast, in the Nearctic realm the vulnerability of FD was significantly higher than expected given the number of threatened species (SES = 3.47, *P* > 0.999, Table 1). A similar trend although not significant was found in the Palearctic realm (SES = 0.84, *P* = 0.66, Table 1). While being the least functionally diverse, the Nearctic and Palearctic realms are the most vulnerable to species loss (Table 1). Although there is a large number of threatened species (743 species accounting for 31% of the Afrotropical fish fauna), the Afrotropical vulnerability is low whereas in the Nearctic and Palearctic realms, fewer species are threatened (147 and 317 species, respectively), but they differ functionally from the rest of the fauna (e.g. sturgeons and eels) and thus lead to a high vulnerability of the functional diversity of this realm. As for alpine plants, tropical trees and coral reef fishes^32^, the threatened species support vulnerable functions, testifying that the imminent extinction of a few endangered species will cause a marked decline of functional diversity across the world faunas.

In addition, when considering only the most threatened species, i.e. those listed as critically endangered (CR), endangered (EN) or vulnerable (VU) by the IUCN, we found that these species account for most of the functional vulnerability in the Nearctic (94.4%) and Oriental (70.5%) realms and to a lower proportion in the Palearctic (51.4%) and even less than 25% in the 3 others realms. In those last realms, most of the vulnerability is supported by endemic species that are potentially threatened although being not evaluated yet (Fig. 4, Table 1). Extending the IUCN evaluation of threats to the world fish fauna is therefore a prerequisite to determine the role played by the most threatened species in the maintenance of the functional diversity over more than half of the world continental areas.

## Conclusion

Assessing functional diversity and its determinants on large scale is pivotal in biodiversity mapping^11^. Indeed, the species number is not a good surrogate for functional diversity as illustrated by the contrasting FD observed here between the two most speciose realms, namely the Neotropics and the Afrotropics. Moreover, FD does not match with functional vulnerability because of the particular functional attributes of threatened species in the Palearctic and Nearctic realms. These mismatches between TD and FD and between FD and functional vulnerability stress the need to consider multiple biodiversity facets in efficient conservation planning. More precisely, future conservation efforts should focus on the vulnerable realms, namely the Nearctic and Palearctic, but also in the Neotropics to protect the habitats hosting the functionally most original species across the world. Such conservation actions should be taken without delay, as the vulnerable functions are overrepresented among the already-listed threatened species. In addition, as the endemic species which status are still unknown also contribute to a high proportion of functional diversity over more than half of the world continental surface, studies at the species level are urgently needed to evaluate the threats they are facing. Considering more specifically freshwater fish, that constitute an emblematic case of massive introductions of non-native species^3^, it is urgent to assess whether the patterns reported here for historical native fish faunas have been blurred by the biotic exchanges between continents for the last centuries. Towards effective assessments of biodiversity-ecosystem services relationships, the large-scale patterns reported here should be completed by local assessments of the key ecological roles played by fish in aquatic ecosystems.

## Methods

### Fish occurrence database

We considered the 6 terrestrial biogeographic realms (Afrotropical, Australian (including Oceania), Nearctic, Neotropical, Oriental and Palearctic) commonly used for freshwater fish^8^,^33^,^34^,^35^. The number and identity of the species occurring in each realm was obtained using the database on freshwater fish occurrences^34^ that contains 9,170 freshwater fish species out of the *ca*. 13,000 strictly freshwater fish species described^30^, and hence covers 77% of the documented world freshwater fish fauna.

### Fish pictures collection

We here developed the most comprehensive fish functional database existing to date. It encompasses 9,170 freshwater fish species out of the *ca*. 13,000 described strictly freshwater fish species (i.e. 77% of the world freshwater fish fauna). We ran an extensive literature review to collect at least one lateral view picture of each species, which were used to measure morphological characteristics (Supplementary Fig. S1a). Some of the pictures were taken on the field by the authors. Fish captures were led in accordance with laws and guidelines concerning live animals, and all the experiments were approved by the Direction of Environment of the French ministry of environment (DEAL), the French Guiana National Park (Parc Amazonien de Guyane), and the research program LABEX CEBA (ANR-10-LABX-25-01).

For all the species present in the taxonomic database we aimed at measuring 10 functional traits describing species strategies for food acquisition and locomotion (Supplementary Fig. S1 and^24^). Fish size was described using the log-transformed maximum body length (values taken from FishBase^36^). In addition to size, 9 morphological traits were measured on side view pictures (Supplementary Fig. S1a) collected during an extensive literature review from more than 200 scientific literature sources including peer-reviewed articles, books and scientific websites. We collected at least one picture (validated photograph or scientific drawing) per species. Only good quality pictures and scientific side view drawings of entire adult animals were kept. Juveniles were not considered as morphological changes can occur during ontogeny. In the event of sexual dimorphism, we only considered male morphology, as female pictures are scarce for most species (especially for Perciforms and Cyprinodontiforms). Using lateral view pictures did not permit to collect as much external morphological information as fresh animals (e.g. oral gape surface, body transversal shape). It would nevertheless be very demanding to collect fresh or museum specimens for more than 9,000 species, and using lateral view pictures was the most efficient way to collect morphological measures for more than 70% of the world freshwater fish fauna.

### Functional traits

For each specimen, 11 morphological measurements were recorded (see Supplementary Fig. S1a) using *ImageJ* software (http://rsb.info.nih.gov/ij/index.html) and were then used to compute 9 unitless ratios describing the morphology of the fish head (including mouth and eye), body, pectoral and caudal fins (Supplementary Fig. S1b). The 10 functional traits (9 unitless ratios and size) selected are commonly used in assessment of fish functional diversity (e.g.^25^,^26^,^37^). Complementary functional traits (e.g. gut length, oral gape area and shape, fecundity) were not included because they are only available for a few species. The quality of the pictures did not allow measurement of all 11 morphological traits in all species, but more than 80% of the species were functionally characterized within each taxonomic order. All nine morphological traits were measured for almost two-thirds of the resulting morphological database (6,030 species) and overall 18% of the morphological measurements were not obtained.

Some species have unusual morphologies (species without tail, flatfishes) that prevent from measuring some morphological traits. We thus defined rules for these few exceptions as in^26^: (*i*) for species with no visible caudal fin (e.g. Sternopygidae, Anguilidae, Plotosidae), Caudal Peduncle Throttling was set to 1 (assuming CFd = CPd, see Supplementary Fig. S1b), (*ii*) for the species with the mouth positioned under the body (e.g. Loricaridae, some Balitoridae such as Gastromyzon) mouth vertical position (Mo) was set to 0, (*iii*) for the species without pectoral fins (e.g. Synbranchiforms and some Anguiliforms) Pectoral fin vertical position (PFl/Bd) was set to 0.; *iv*) for flatfishes Bd was the body width as the fish lies on one side of its body. We hence assumed that Pleuronectiforms are functionally closer to dorso-ventrally flattened fishes (e.g. Gastromyzon) than to laterally compressed fishes (e.g. Symphysodon).

Other traits, such as ecological, behavioural or physiological traits were not considered in this study, because there is currently no database available at the world scale.

### Functional space

The 10 functional traits were ordered in a multidimensional functional space by means of Principal Components Analysis using a regularized algorithm designed for ordination analysis to handle the missing values^38^. The first 5 axes account for 80.5% of the total variance (each selected axis had an eigenvalue >1) (Supplementary Fig. S4) and were retained to build a 5-dimensional functional space. We assessed the robustness of our findings using sensitivity procedure. We tested the effect of trait identity on the functional distance between species by rerunning all analyses using all combinations of nine functional traits out of ten. The results were hardly affected by this procedure (Mantel tests *r* > 0.900, *P* < 0.001). Thus, our findings are not affected by functional trait selection.

### Threatened species

Two types of threatened species were considered, the “most threatened” and the “potentially threatened”. The “most threatened” species were identified as the species listed as critically endangered (‘CR’), endangered (‘EN’) and vulnerable (‘VU’) in the most recent IUCN Red List assessment^27^. The IUCN Red List of threatened species although being the most objective and authoritative system for classifying species in terms of the risk of extinction at the global scale, remains affected by spatial disparities in species evaluation. To deal with incompleteness of the IUCN assessment (52.5% of the species occurring in our database have not been IUCN evaluated, see Supplementary Table S1), strictly endemic species (species occurring in only one drainage basin across the world^28^,^30^,^34^) which IUCN status was not “near threatened (NT)” nor “least concern (LC)” were considered as “potentially threatened”. Those species with limited spatial distribution have been recognized as prone to extinction following human disturbance^4^,^28^,^39^. Thus, a total of 3,443 (37.5%) species out of the 9,170 species were considered as threatened.

### Biodiversity indices

Taxonomic diversity (TD) was calculated as the number of species in each realm (or in each order). Functional diversity (FD) was assessed with the functional richness index (FRic^40^), computed as the volume of the minimum convex hull that includes all the species in the 5-dimensional functional space. The higher the FD, the higher the richness of combinations of functional trait values in the species pool considered. FD and TD indices were computed for all species in each realm, and for all orders within each realm.

### Statistical analyses

To test whether observed functional diversity of a realm was significantly different from functional diversity of a random subset of species, we used null models based on randomization of species pools. To simulate a realistic pool of species in each realm, the number of species per taxonomic order was kept constant in the random choice process (999 iterations). The standardized effect size (*SES*) was used to measure the difference between observed values (obs) and null expectation (rand): *SES* = (FD_obs_ − mean (FD_rand_))/sd (FD_rand_). The significance of the difference from null expectations was tested using a two-tailed test (∝ < 0.05). When FD_obs_ is lower than 97.5% of FD_rand_ values, the assemblage is considered as functionally clustered. When FD_obs_ is greater than 97.5% of FD_rand_ values, the assemblage is considered as functionally overdispersed.

The dissimilarity in FD and TD between realms was measured as taxonomic and functional turnover^41^. Taxonomic turnover measures the replacement of species between two assemblages independently of the difference in species richness^41^. Similarly, functional turnover measures the replacement of functional volume independently of the difference in functional richness. Taxonomic and functional turnover were calculated using the same formula: 
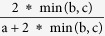
, where *a* is the number of species (or the volume) shared by the two assemblages and *b* and *c* the number of unique species (or the unique volume) hosted by the two assemblages^41^,^42^. Both taxonomic and functional turnover varies from 0, when the two assemblages are completely similar, to 1 when the two assemblages shared no species (taxonomic) or no portion of the functional space.

The functional uniqueness of each fish order *o* in each realm *r* was computed as the proportion of the functional space filled only by the order considered: 
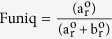
 , where *a*_*r*_^*o*^ is the volume filled only by focal order *o* and *b*_*r*_ is the volume shared with all other orders present in realm *r*. Taxonomic and functional turnover, and F_uniq_ were computed using the ‘functional.beta.core’ function from the ‘betapart’ R package^43^.

The functional vulnerability (FV) to threatened species loss in each realm was computed as the proportion of the FD of the realm that will remain if all threatened species got extinct: FV_obs_ = (FD_obs_ − FD_not threatened_)/FD_obs_. Functional vulnerability is null when species not threatened contribute fully to the FD of the realm and tends to the 100% if all the most extreme combinations of traits are supported only by threatened species.

To test the significance of the observed functional vulnerability given the number of threatened species we designed a null model based on a random choice of the species expected to be lost (999 iterations). The standardized effect size (SES) was used to measure the difference between predicted FD loss values due to extinction of threatened species (FV_obs_) and null expectation of FD loss (rand): SES = (FV_obs_ − mean(FV_rand_))/sd (FV_rand_). The significance of the difference from null expectations was tested using a two-tailed test (∝ < 0.05). A p-value lower than 0.025 indicates that threatened species are functionally distinct and over-contribute to FD whereas a p-value higher than 0.975 indicates that threatened species are redundant with other species of the realm FD.

We then ran the same analyses considering only the most threatened species, i.e. those listed as “critically endangered”, “endangered” or “vulnerable” by the IUCN^27^.

All statistical analyses were performed with R software version 3.0^44^.

## Additional Information

**How to cite this article**: Toussaint, A. *et al*. Global functional diversity of freshwater fish is concentrated in the Neotropics while functional vulnerability is widespread. *Sci. Rep*. **6**, 22125; doi: 10.1038/srep22125 (2016).

## Supplementary Material

Supplementary Information

## Figures and Tables

**Table 1:** Functional Vulnerability (FV) supported by the threatened species in the six biogeographic realms.

	Threatened species					Most threatened species				
	Nb. of species	FV (%)	SES	*P* value		Nb. of species	FV (%)	SES	*P* value	
Afrotropical	743	14.12	−0.85	0.26	ns	**356**	3.73	−0.91	0.12	ns
Australian	62	7.47	−0.91	0.21	ns	**40**	1.66	−1.03	0.12	ns
Nearctic	147	**43.56**	**3.47**	**>0.999**	***	117	**41.15**	**4.05**	**>0.999**	***
Neotropical	1680	**9.03**	**−2.32**	**<0.001**	***	52	1.67	0.74	0.91	ns
Oriental	350	9.11	−0.84	0.21	ns	151	7.2	0.31	0.71	ns
Palearctic	317	33.46	0.84	0.66	ns	108	17.29	1.02	0.88	ns

For each realm, we computed the vulnerability of FD to the loss of threatened species as the proportion of the FD of the realm that will remain if all threatened species got extinct (see details in Methods). In each realm, the number of threatened species is indicated (Nb. of species). Two levels of threat were considered. The “threatened species” are the species listed as CR, EN and VU by IUCN, and the endemic species (i.e. species occurring in only one drainage basin across the world) not listed as “near threatened” nor “least concern” by the IUCN. The “Most threatened species” are only the species listed as CR, EN and VU by IUCN. The observed vulnerability value was compared to the expected vulnerability given number of threatened (or most threatened) species in each realm, i.e. 999 values of FD loss simulated with a random extinction of the same number of species than the number actually listed as threatened (or most threatened) in the realm. Standardized Effect Size (SES) was calculated as the difference between the observed loss in FD and the mean of the random loss of FD divided by the standard error of the random loss of FD. A negative SES means that the observed loss of FD is higher (i.e. more negative) than expected if extinctions were random whereas a positive SES means that the loss of FD observed is lower than expected. The significance of the difference from null expectations was tested using a two-tailed test (α < 0.05). *ns*: non-significant; *p < 0.025 or *p* > 0.975; **p < 0.01 or *p* > 0.99; ***p < 0.001 or *p* > 0.999. The FV values significantly different from the null ex*p*ectation are in bold font.
