# Supplementary material for: Global functional diversity of freshwater fish is concentrated in the Neotropics while functional vulnerability is widespread
Source: Sci Rep. 2016 Mar 16;6:22125. doi: 10.1038/srep22125 (PMC4793233; doi:10.1038/srep22125)
Supplement: Supplementary Information [file srep22125-s1.docx]

**Global functional diversity of freshwater fish is concentrated in the Neotropics while functional vulnerability is widespread**

Toussaint A. ^1*^, Charpin N. ^1^, Brosse S. ^1§^ & Villéger S. ^2§^

**SUPPLEMENTARY INFORMATION**

^1^CNRS, UPS, ENFA, UMR 5174 EDB (Laboratoire Évolution et Diversité Biologique), Université Paul Sabatier, 118 route de Narbonne, F-31062 Toulouse, France

^2^Laboratoire Biodiversité Marine et ses Usages (MARBEC), UMR 9190 CNRS-UM-IFREMER-IRD, Université de Montpellier, CC 093, F-34095 Montpellier Cedex 5, France

^§^ Co-senior authorship

*Corresponding author: Aurèle Toussaint

E-mail: aurele.toussaint@univ-tlse3.fr

Tel: +33 561556747

Fax: +33 561557327

Address: Laboratoire Evolution et Diversité Biologique (UMR 5174), Université Paul Sabatier, 118 Route de Narbonne, 31062 Toulouse Cedex 4, France

**Figure S1. Morphological measurements (a) and functional traits (b) measured on each fish species**


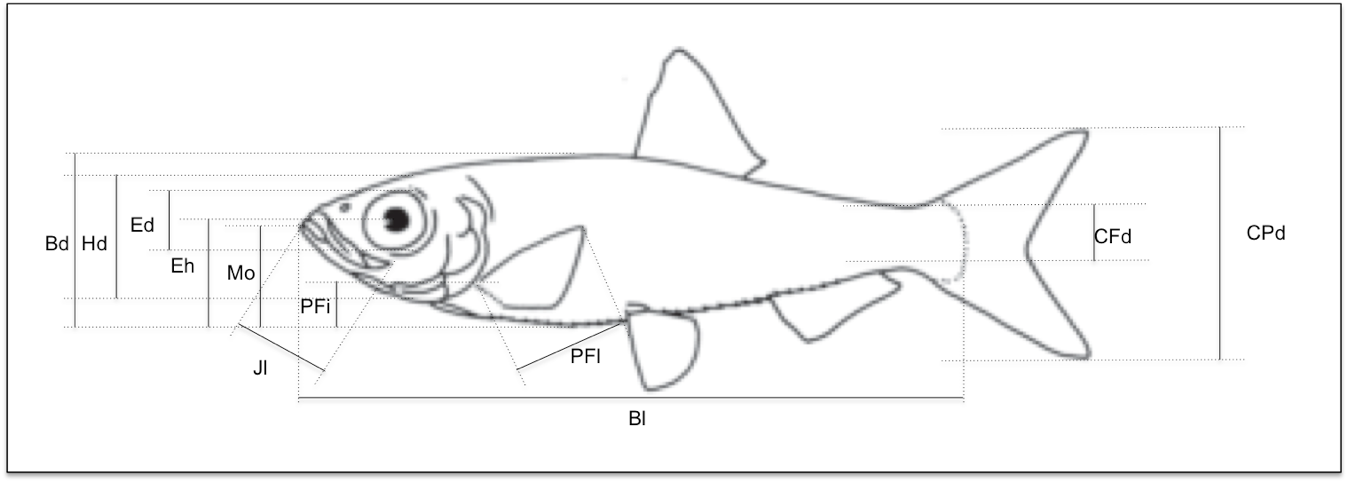


**a. Morphological measurements**

|  | | |
| --- | --- | --- |
| Code | Name | Protocol for measurement |
| Blmax | Maximum Body length | Maximum adult length |
| Bl | Body length | Standard length (snout to caudal fin basis) |
| Bd | Body depth | Maximum body depth |
| Hd | Head depth | Head depth at the vertical of eye |
| CPd | Caudal peduncle depth | Minimum depth of the caudal peduncle |
| CFd | Caudal fin depth | Maximum depth of the caudal fin |
| Ed | Eye diameter | Vertical diameter of the eye |
| Eh | Eye position | Vertical distance between the centre of the eye to the bottom of the body |
| Mo | Oral gape position | Vertical distance from the top of the mouth to the bottom of the body |
| Jl | Maxillary jaw length | Length from snout to the corner of the mouth |
| PFl | Pectoral fin length | Length of the longest ray of the pectoral fin |
| PFi | Pectoral fin position | Vertical distance between the upper insertion of the pectoral fin to the bottom of the body |
| All measurements were made on pictures except Blmax values, which were downloaded from Fishbase.org | | |

| **b. Functional traits** | | |
| --- | --- | --- |
| **Functional traits** | **Formula** | **Potential link with fish functions** |
| Body length | *BLmax* | Size is linked to metabolism, trophic impacts, locomotion ability, nutrient cycling |
| Body elongation | $\frac{Bl}{Bd}$ | Hydrodynamism  Position of fish and/or of its prey in the water column |
| Eye vertical position | $\frac{Eh}{Bd}$ |  |
| Relative Eye size | $\frac{Ed}{Hd}$ | Visual acuity |
| Oral gape position | $\frac{Mo}{Bd}$ | Feeding position in the water column |
| Relative maxillary length | $\frac{Jl}{Hd}$ | Size of mouth and strength of jaw |
| Body lateral shape | $\frac{Hd}{Bd}$ | Hydrodynamism and head size |
| Pectoral fin vertical position | $\frac{PFi}{Bd}$ | Pectoral fin use for swimming |
| Pectoral fin size | $\frac{PFl}{Bd}$ | Pectoral fin use for swimming |
| Caudal peduncle throttling | $\frac{CFd}{CPd}$ | Caudal propulsion efficiency through reduction of drag |

**Figure S2. Fish functional diversity in the 6 biogeographic realms for the first 5 PCA axes.** The functional space filled by all the species of a realm is delimited by the dashed black line. The colored polygons within it show the functional richness of the 8 main fish orders. The white area represents the space filled by the global pool of freshwater fish species. The percentage of variance explained by each axis is given in brackets (see also Figure S4).

(a) PC axes 3 and 4.

(b) PC axes 4 and 5.

**Figure S3. An overview of the Neotropical fish functional diversity.** The biplot at the center of the figure shows fish position (red dots) in the two first axes plane of the functional space (see Fig. 1 for details). The FD of the world fish fauna is in white and that of the Neotropical realm is in blue. Numbers indicate fish species (with order and familly in brackets) as follows: 1- *Awaous flavus* (Perciforms, Gobiidae); 2- *Cynodon meionactis* (Characiforms, Cynodontidae); 3- *Hemiancistrus medians* (Siluriforms, Loricariidae); 4- *Doras carinatus* (Siluriforms, Doradidae); 5- *Sturisoma aureum* (Siluriforms, Loricariidae); 6- *Guianacara geayi* (Perciforms, Cichlidae) ; 7- *Serrasalmus rhombeus* (Characiforms, Serrasalmidae); 8- *Potamorrhaphis guianensis* (Beloniforms, Belonidae); 9- *Ituglanis amazonicus* (Siluriforms, Trichomycteridae) ; 10- *Sternopygus macrurus* (Gymnotiforms, Sternopygidae) ; 11- *Crenicichla johanna* (Perciforms, Cichlidae) ; 12- *Boulengerella lucia* (Characiforms, Ctenoluciidae); 13- *Moenkhausia oligolepis* (Characiforms, Characidae); 14- *Cyphocharax abramoides* (Characiforms, Curimatidae). All photos by S. Brosse, except 1 (R. Macieira), 5 (R. Bartz, M. Aka, M. Freak) and 14 (D. Torres Hashiguti de Freitas). Fish pictures were taken during field works that complied with national laws and were approved by relevant licensing and ethical committees. The ‘Sturisoma is a genus of catfishes (order Siluriformes) of the family Loricariidae’ ([https://fr.wikipedia.org/wiki/Sturisoma_aureum#/media/File:Sturisoma_aureum_Richard_Bartz.jpg](https://fr.wikipedia.org/wiki/Sturisoma_aureum)) is licensed under the Attribution-Share-Alike 3.0. Unported license. The license terms can be found on the following link: <http://creativecommons.org/licenses/by-sa/2.5/>


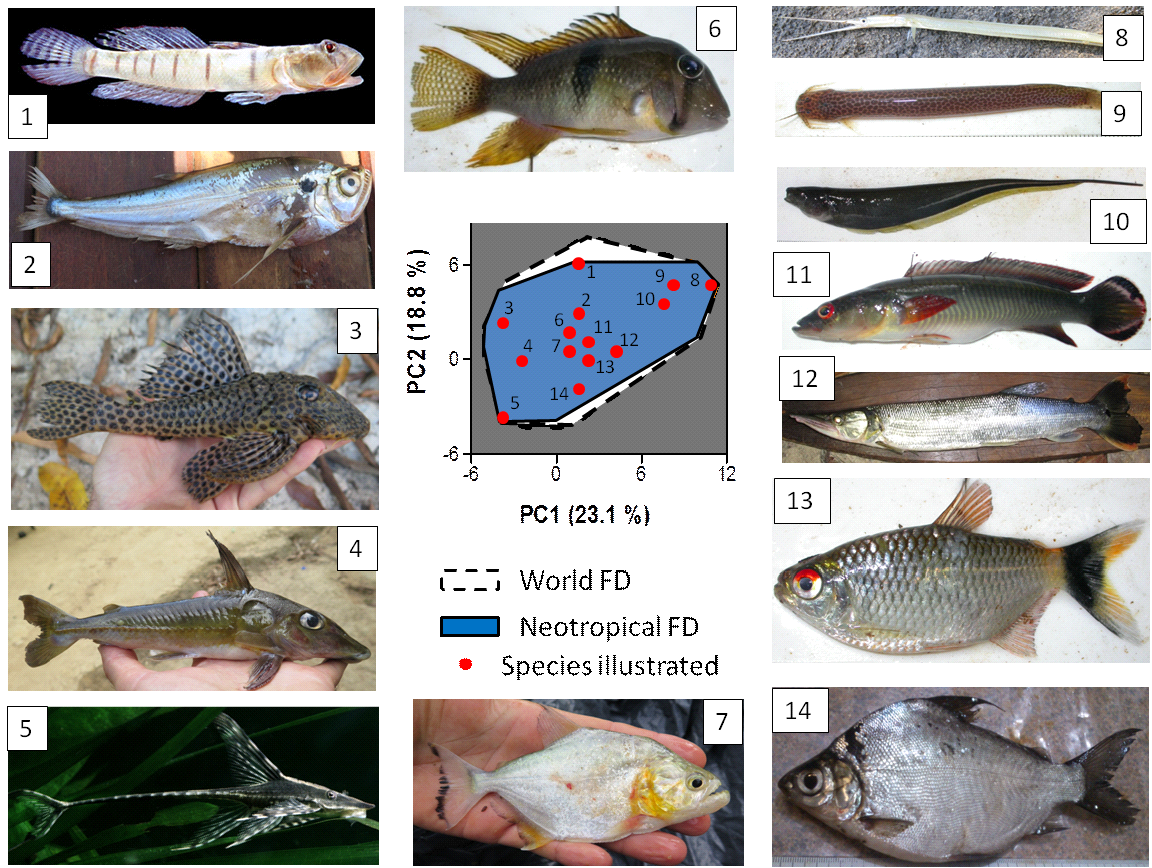


**Figure S4. Results of the Principal Components Analysis on functional traits.** A. Eigenvalues and percentages of variance explained by each axis. B. Percentage of contribution of each functional trait to each axis.

| A. | | | | | |
| --- | --- | --- | --- | --- | --- |
|  | Axis 1 | Axis 2 | Axis 3 | Axis 4 | Axis 5 |
| Eigenvalues | 2.31 | 1.88 | 1.60 | 1.23 | 1.03 |
| Percentage of variance | 23.1 | 18.8 | 16.0 | 12.3 | 10.3 |
| Cumulative percentage of variance | 23.1 | 41.9 | 57.9 | 70.2 | 80.5 |

| B. | | | | | |
| --- | --- | --- | --- | --- | --- |
|  | Axis 1 | Axis 2 | Axis 3 | Axis 4 | Axis 5 |
| Relative Eye size | 5.7 | <1 | **26.8** | **16.3** | 7.4 |
| Oral gape position | **25.4** | 3.6 | 7.7 | 3.02 | <1 |
| Relative maxillary length | **20.4** | 5.3 | <1 | <1 | 2.0 |
| Eye vertical position | 11.0 | 13.6 | **15.5** | 2.2 | <1 |
| Body elongation | 6.8 | 11.6 | **16.4** | **18.3** | <1 |
| Body lateral shape | <1 | 13.4 | **29.3** | 8.8 | 2.4 |
| Pectoral fin vertical position | **23.9** | 4.8 | <1 | 3.4 | 1.9 |
| Pectoral fin size | 5.2 | 24.0 | <1 | 11.1 | 4.2 |
| Caudal peduncle throttling | <1 | 3.5 | <1 | 1.9 | **80.6** |
| Maximum body length | <1 | **19.4** | 3.4 | **34.9** | <1 |
| The contribution of each functional trait to the first 5 axes is expressed as percentages and values higher than 15% are in bold font. | | | | | |

**Table S1: Number of species for each IUCN threat level.** CR: critically endangered, EN: endangered, VU: vulnerable, NT: near threatened, LC: least concern, DD: data deficient, NE: not evaluated)

|  | CR | EN | VU | NT | LC | DD | NE |
| --- | --- | --- | --- | --- | --- | --- | --- |
| Afrotropical | 19 | 39 | 85 | 27 | 434 | 102 | 1622 |
| Australian | 0 | 4 | 10 | 4 | 29 | 6 | 328 |
| Nearctic | 14 | 24 | 25 | 7 | 242 | 47 | 333 |
| Neotropical | 3 | 8 | 23 | 4 | 95 | 23 | 3685 |
| Oriental | 30 | 37 | 82 | 28 | 379 | 83 | 936 |
| Palearctic | 19 | 21 | 34 | 7 | 186 | 51 | 673 |
